# Evaluation of Anticancer and Immunomodulatory Effects of Microwave-Extracted Polysaccharide from *Ruditapes philippinarum*

**DOI:** 10.3390/foods13223552

**Published:** 2024-11-07

**Authors:** Mengyue Liu, Fei Li, Shuang Feng, Jiamin Guo, Jia Yu, Shengcan Zou, Xiang Gao, Yuxi Wei

**Affiliations:** 1 College of Life Sciences, Qingdao University, Qingdao 266071, China; lmy02112021@163.com (M.L.); pdlifei@163.com (F.L.); xx526916212@126.com (X.G.); yuxiw729@163.com (Y.W.); 2 Qingdao Yihai Industry Holdings Co., Ltd., Qingdao 266105, China; m18565444972@163.com (S.F.); gjmstudy@163.com (J.G.); zoushengcan@chenland.cn (S.Z.); 3 Qingdao Chenlan Pharmaceutical Co., Ltd., Qingdao 266105, China

**Keywords:** *Ruditapes philippinarum* polysaccharide, antitumor, immunomodulatory functions, antioxidant activities

## Abstract

In recent years, research on active polysaccharides has progressed significantly, particularly regarding their anticancer and immunomodulatory properties. Among these, clam polysaccharides, a type of marine-derived polysaccharide, exhibit notable biological activities, including both anticancer effects and immune modulation. The aims of this study are to investigate the anticancer and immunomodulatory effects of microwave-extracted clam polysaccharide (MCP) in vitro. Cell experiments demonstrated that MCP significantly inhibited both colony formation and migration of HT-29 cells. Furthermore, treatment with MCP led to the downregulation of Bcl-2 gene expression, a reduction in mitochondrial membrane potential, activation of cytochrome C gene and caspase-3 gene, and, finally, the induction of apoptosis in HT-29 cells, implying the involvement of the mitochondrial pathway. Additionally, MCP was found to prompt a phenotypic shift in macrophages from M2 to M1 subtype and from M0 to M1 subtype. MCP also decreased reactive oxygen species (ROS) levels within the cancer cells, thereby augmenting anticancer efficacy through a dual mechanism of immune activation and antioxidant enhancement. These findings suggest that MCPs present significant potential as natural antitumor agents and immunomodulators, especially in the development of functional foods or drugs.

## 1. Introduction

In the past decades, the incidence of adult colorectal cancer (CRC) has been steadily increasing, primarily attributed to detrimental dietary habits [1]. Currently, the primary treatment for CRC involves surgery, often combined with radiotherapy or chemotherapy. Conversely, the conventional treatment is frequently associated with severe side effects and is poisonous to healthy cells [2]. Recently, immunotherapy has become an effective new treatment option for CRC [3]. Despite its potential, immunotherapy is not universally effective due to variations in the patients’ immune backgrounds, primarily influenced by different CRC subtypes [4].

Natural anticancer products and immune regulators, particularly many natural polysaccharides, have garnered widespread attention. For example, LNT (*lentinoglycan* polysaccharide) has been shown to significantly improve the cancer response of advanced gastric cancer [5], and RRTP80-1 (*Rosa roxburghii* polysaccharide) can markedly reduce the size of A549 transplanted tumors in zebrafish [6]. Recent research has focused on active polysaccharides extracted from marine organisms, which have captivated scientists globally. *Mollusca*, one of the biggest groups of marine organisms, inhabiting diverse environments in the ocean (shores or abyssal zones), are of particular interest [7]. Polysaccharides derived from various classes of the *Phylum Mollusca* are noted for their unique properties and reported to exhibit a range of beneficial properties, including the anticoagulant activity of fucosylated chondroitin sulfate polysaccharide from sea cucumber [8], anticancer activity of OP-1 (oyster polysaccharide) [9] and AVP (abalone polysaccharide) [10], immunomodulatory activity of MSGA (sea urchin polysaccharide) [11] and CSP-1 (*Cyclina sinensis* polysaccharide) [12], antioxidant of SF (starfish polysaccharide) [13] and others. The bioactivity of polysaccharides is widely recognized, prompting ongoing improvements in extraction methods [14]. In particular, microwave-assisted extraction utilizes microwave radiation to generate substantial heat, which causes the disruption of cell membranes and walls, thereby facilitating the release of polysaccharide components. This method not only shortens reaction times but also enhances overall yields [15].

*Ruditapes philippinarum* (clam) is a prominent species among Mollusca and a valuable edible bivalve in China’s aquaculture sector. It is widely distributed along the coasts of China, Korea, and Southeast Asia [16]. In China, the annual yield of *R. philippinarum* accounts for about 90% of all global production [17]. In addition to its significant nutritional value, *R. philippinarum* is an important ingredient in traditional Chinese medicine, having the effects of clearing phlegm and reducing inflammation [18]. Research on *R. philippinarum* has predominantly focused on polypeptides [19,20], transcriptomic analyses [21,22], and the toxicity of nanomaterials in marine model biological studies [23,24]. A previous study indicated that the glycoprotein coagulant RpCTL from *R. philippinarum* is related to immune responses and plays a role in the immune defense mechanisms of *R. philippinarum* [17]. However, the potential anticancer and immunomodulatory bioactivities of polysaccharides derived from *R. philippinarum* have not been so thoroughly investigated.

This study aimed to evaluate the anticancer and immunoregulatory activity of microwave-extracted polysaccharide from *R. philippinarum* (MCP). This study will offer new insights into the potential utilization of MCP as a natural ingredient in functional foods or pharmaceuticals for anticancer and immunoregulatory applications.

## 2. Materials and Methods

### 2.1. Materials

Fresh clams (*Ruditapes philippinarum*) were acquired from a local market (Qingdao, China). All cell lines were acquired from the Resource Center at the Shanghai Institute of Life Sciences, Chinese Academy of Sciences (Shanghai, China). DMEM/high glucose medium was purchased from Hyclone (Logan, UT, USA), while McCoy’s 5A and RPMI-1640 medium were acquired from Pricella Biotechnology (Wuhan, China). Fetal bovine serum (FBS) was sourced from Gibco Life Technologies (Grand Island, NY, USA), and lipopolysaccharide (LPS) and interferon-γ (INF-γ) were supplied by Sigma (St. Louis, MO, USA). ELISA kit for the analysis of CD86, CD206, CD163, TNF-α, iNOS, and IL-10 were obtained from Cloud-Clone Corp (Wuhan, China). Trypsin-EDTA solution, penicillin-streptomycin liquid, and dimethyl sulfoxide (DMSO) were purchased from Solarbio (Beijing, China). Annexin V-fluorescein isothiocyanate (FITC)/propidium iodide (PI) apoptosis detection kit, CCK-8 kit, Neutral red kit, and mitochondrial membrane potential assay kit were obtained from Beyotime Biotechnology (Shanghai, China). All other chemical reagents were of analytical grade.

### 2.2. Sample Preparation of MCP

Fresh clam meat paste was weighed and mixed with water at a solid–liquid ratio of 1:7. The mixture was transferred to a microwave-safe container, and heated in a microwave oven for 4 min. After heating, the mixture was centrifuged (4000 rpm, 20 min) to collect the supernatant. The supernatant was collected and the pellets were discarded. Next, 95% ethanol was slowly added while stirring to the collected supernatant, until the final ethanol concentration of the mixture reached 80%. The samples were then stored at 4 °C for 12 h; centrifugation was carried out at 10,000 rpm for 10 min to collect the precipitate. This ethanol precipitation process was repeated once. The collected precipitates were then freeze-dried using a freeze-dryer. Following ultrafiltration, polysaccharides with a molecular weight exceeding 50 kDa were selected, resulting in the isolation of the polysaccharide named as MCP. The total sugar content of MCP was determined to be 80.7% using the phenol-sulfuric acid method. The MCP, as determined by the PMP-HPLC method, was primarily composed of glucose, arabinose, rhamnose, glucuronic acid, galacturonic acid, and mannose (Figure S1). The relative proportions of these components are detailed in Table S1.

### 2.3. Cell Culture

MCG-803 (human gastric cancer cells), HepG-2 (human hepatocellular carcinoma), HuH-7 (human hepatocellular carcinoma), A549 (human non-small cell lung cancer cells), RAW 264.7 cells (mouse mononuclear macrophages cells) were cultured in high-glucose DMEM containing 10% fetal bovine serum and 1% double antibody (penicillin-streptomycin). HT-29 cells (human colorectal carcinoma cells) were cultured in McCoy’s 5A with 10% fetal bovine serum and 1% double antibody. Some 4T1 (mouse breast carcinoma cells), B16 (mouse melanoma cells), CT-26 (mouse colon cancer cells) cells were cultured in RPMI-1640 complete medium. Both HT-29 and CT-26 are colorectal cancer cell lines, although they originate from different species. HT-29 cells are of human colorectal origin, while CT-26 cells are derived from mouse colorectal tissue.

### 2.4. Anticancer Activity of MCP

#### 2.4.1. Tumor Cytotoxicity Experiment

The toxicity of MCP was assessed in eight types of tumor cells (MCG-803, HepG-2, HuH-7, A549, HT-29, CT-26,4T1 and B16) using a CCK-8 assay. To summarize, tumor cells were plated in a 96-well dish at a density of 5 × 10^5^ cells/mL. The group receiving medium devoid of polysaccharides acted as the control. MCP was added to the cells at five concentrations (250, 500, 1000, 1500 and 2000 µg/mL) on the 96-well plate. The cells were incubated for 48 h. Cell viability was calculated using the CCK-8 kit with absorbance measured at 450 nm by a multi-function microplate reader (Tecan, Grödig, Austria)

Cell viability (%) = (A_drug_ − A_blank_)/(A_control_ − A_blank_) × 100 (1)

where A_drug_ is the absorbance value of the cell groups treated with MCP, A_control_ is the absorbance readings from the cell groups exposed to the complete medium and A_blank_ is the absorbance of the complete medium in the absence of cells.

#### 2.4.2. Colony Formation

To evaluate the effect of MCP on the proliferative activity of HT-29 single cells, cells were incubated with MCP (0, 250, 500, 1000 and 2000 μg/mL) for 24 h, after reseeding with 5 × 10^2^/well into a 6-well plate. The medium was replaced with McCoy’s 5A medium every three days until day 8. On the last day, cells were washed with PBS, fixed with polyoxymethylene (4%) and stained with crystal violet solution (0.5%) for 10 min. The cell colonies were photographed using Amersham Imager (AI680RGB, GE, Tokyo, Japan) and quantified using Fiji is just ImageJ software (version 2.7.0).

#### 2.4.3. Wound Healing Assay

The HT-29 cells were seeded in a 6-well plate at a density of 2 × 10^5^ cells per well, using a culture medium containing 10% FBS. After an overnight incubation, cells were carefully scratched with 200 μL micro pipette tips and exposed to media containing MCP for periods of 12, 24, and 48 h, respectively. The wounded area in each group was observed under a microscope and the scratched area was quantified using ImageJ software as previously described.

Cell Migration Rate (%) = (W_0_ − W_t_)/W_0_ × 100 (2)

where W_0_ represents the initial wound width (the width measured prior to cell migration), and W_t_ denotes the wound width measured after a specified period of time.

#### 2.4.4. Analysis of Cell Apoptosis

Cell apoptosis triggered by MCP was evaluated through the PI-Annexin V-FITC double-staining technique. HT-29 cells were set to a concentration of 5 × 10^5^ cells/mL and seeded into 6-well plates. The cells were subsequently incubated with varying concentrations of MCP or without MCP. After a 48 h incubation, the cells were treated with 5 μL of Annexin V-FITC and PI for 20 min in a dark environment. The stained cells were subsequently analyzed using a flow cytometer. (BD FACS Celesta, Becton Dickinson, Franklin Lakes, NJ, USA).

#### 2.4.5. Detection of Mitochondrial Membrane Potential (Δ*ψm*)

Mitochondrial membrane potential (Δ*ψm*) was assessed using the JC-1 fluorescence probe. In summary, HT-29 cells underwent pre-treatment as previously detailed. Following this, the cells were suspended in 0.5 mL of PBS and incubated with the JC-1 staining solution at 37 °C for 20 min, according to the mitochondrial membrane potential staining kit protocol. Finally, the fluorescence of the cells was analyzed using a flow cytometer.

Furthermore, HT-29 cells underwent pre-treatment as previously detailed. The cells were then examined using the inverted fluorescence microscope (Axio Vert.A1, Carl Zeiss AG, Oberkochen, Germany).

#### 2.4.6. Quantitative Real-Time Reverse Transcription Polymerase Chain Reaction (RT-qPCR)

Briefly, HT-29 cells were pretreated as described above. Total RNA was extracted from treated samples using RNA Quick Extraction Kit. The ChamQ universal SYBR QPCR premix, primer, and cDNA template were mixed in a specific proportion. Quantitative PCR was carried out on the CFX Connect TM Real-Time PCR System (Bio-Rad, Hercules, CA, USA). The PCR primer sequences are shown in Table 1. Relative quantification of the target genes was calculated based on the internal reference β-actin according to the 2^−ΔΔCt^ method.

### 2.5. Immunomodulatory Activity of MCP

#### 2.5.1. Immune Cell Viability of MCP

The CCK-8 assay was utilized to evaluate the proliferative effects of MCP on the mouse macrophage line RAW 264.7. In brief, immunity cells were inoculated into a 96-well plate at a density of 5 × 10^5^ cells/mL, utilizing a medium devoid of polysaccharides served as the control group. MCP was added to the cells at five concentrations (50, 100, 150, 200, 250, 300 and 1000 µg/mL) on the 96-well plate. A combination of LPS (100 ng/mL) and INF-γ (20 ng/mL) or IL-4 (20 ng/mL) was used as a model control. Cell viability was calculated using the CCK-8 kit by assessing the absorbance at 450 nm with a Multi-Function Microplate Reader.

#### 2.5.2. Phagocytosis Ability Effect of MCP

The phagocytic ability of macrophages was detected using neutral red staining. Following the cell culture protocols outlined above, the cells were incubated for 24 h. Next, neutral red solution was added using the neutral red kit and incubated for 2 h. After incubation, the supernatant was carefully removed, and the 96-well plates underwent three washes with PBS to eliminate any residual neutral red. Next, 200 μL of cell lysates were added to each well, and the plates were agitated for 10 min to guarantee thorough cell lysis. Eventually, the absorbance was recorded at 540 nm using a multi-function microplate reader.

#### 2.5.3. Secretion of Macrophage M1 and M2 Markers

RAW 264.7 macrophages were subjected to treatment with MCP (200 or 300 μg/mL), IL-4 (20 ng/mL) or a combination of LPS (100 ng/mL) and INF-γ (20 ng/mL) for 24 h. RAW 264.7 macrophages were treated with IL-4 (20 ng/mL) for 12 h to induce the M2 phenotype. Then, the M2 macrophages were treated with MCP at various concentrations (200 or 300 μg/mL) for an additional 24 h. The levels of CD86, TNF-α, iNOS, CD206, CD163 and IL-10 were measured using ELISA.

#### 2.5.4. Transwell Co-Culture System of RAW 264.7 and CT-26

Tumor microenvironment simulation was conducted using Transwell chambers (Labselectl, 14111, Hefei, China) with polycarbonate (0.4 μm pore size, 24 mm diameter). RAW 264.7 cells (2 × 10^4^ cells/mL of medium) were filled with 1 mL of DMEM containing 10% FBS at the upper chamber. CT-26 cells (1 × 10^5^ cells/mL of medium) were filled with 1 mL of DMEM containing 10% FBS at the lower part of the chamber. Both RAW 264.7 cells and CT-26 cells were incubated to adhere for 2 h under 5% CO_2_ and 37 °C. After incubation, the upper and lower chambers were then combined together and an appropriate amount of medium was added to each chamber. MCP (200, 300 μg/mL of medium) was then added to the upper chamber.

### 2.6. Antioxidant Activities

#### 2.6.1. ABTS^•+^, DPPH•, and Hydroxyl Free Radical Scavenging Analysis

The ability of MCP to scavenge ABTS^•+^, DPPH•, and hydroxyl radicals was assessed, using vitamin C (Vc) as the reference standard. MCP was dissolved at the concentrations of 100, 200, 400, 600, 800, 1000, 2000 and 4000 μg/mL, while Vc was tested at concentrations of 10, 20, 40, 60, 80, 100, 200, 400, 600, 800, 1000, 2000, 4000 mg/mL. The DPPH• and hydroxyl radical assay was detected by the Nanjing Jian Cheng Kit (Nanjing, China).

For ABTS^•+^ radical assay, a 7 mmol/L ABTS^•+^ solution and a 2.45 mmol/L K_2_S_2_O_4_ solution were mixed well and incubated in the dark for 16 h. The solution obtained was subsequently diluted using a 5 mmol/L phosphate buffer (PBS, PH = 7.4) to reach an absorbance value of approximately 0.70 at 734 nm. The sample, along with the positive control and blank groups, were combined with the ABTS^•+^ working solution in a 1:4 ratio. MCP mixed with PBS served as the control for the sample. These solutions were stored in the dark at room temperature for 10 min prior to measuring the absorbance at 734 nm.

The percentage of inhibition was determined using the following formula:

Inhibition rate (%) = [1 − (A – A_0_)/A_x_] × 100% (3)

where A, A_0_, and A_x_ represent the absorbance values of the sample or Vc (sample or Vc combined with the ABTS^•+^ working solution), the blank (distilled water with the ABTS^•+^ working solution), and the sample control (sample mixed with PBS), respectively. The EC_50_, defined as the concentration of the sample required to achieve 50% radical scavenging, can be determined through linear regression analysis based on the relationship between the percentage of scavenging and the concentration of the sample.

#### 2.6.2. Intracellular Reactive Oxygen Species (ROS) Detection

The fluorescein-labeled dye 2′,7′-Dichlorodihydrofluorescein diacetate (DCFH-DA) (Beyotime, Shanghai, China) was used to measure intracellular ROS levels. DCFH-DA can penetrate cell membranes and is hydrolyzed to DCFH by intracellular esterase. The reduced form of DCFH is susceptible to oxidation by ROS, resulting in the formation of highly fluorescent 2′,7′-dichlorofluorescein (DCF). Briefly, HT-29 cells were pretreated as described above. The supernatant was discarded after three washes with PBS, the cells were resuspended in a medium containing the DCFH-DA probe. The cells were then incubated for 20 min at 37 °C in the dark. Following this, the cells were washed and resuspended in 500 μL PBS. The intracellular ROS levels were determined by measuring the fluorescence intensity of the cells using flow cytometry.

### 2.7. Statistical Analysis

Figures were created using GraphPad Prism 8.2.1 software, and the descriptive statistics are presented as the mean ± standard deviation (SD). The variance among the groups was evaluated using a one-way ANOVA conducted with SPSS version 21.0. Values of *p* < 0.05 and *p* < 0.01 were considered statistical significance and highly significant differences, respectively.

## 3. Results

### 3.1. Antitumor Activity of MCP In Vitro

#### 3.1.1. Effect of MCP on the Viability of Different Cells

**Figure 1:**
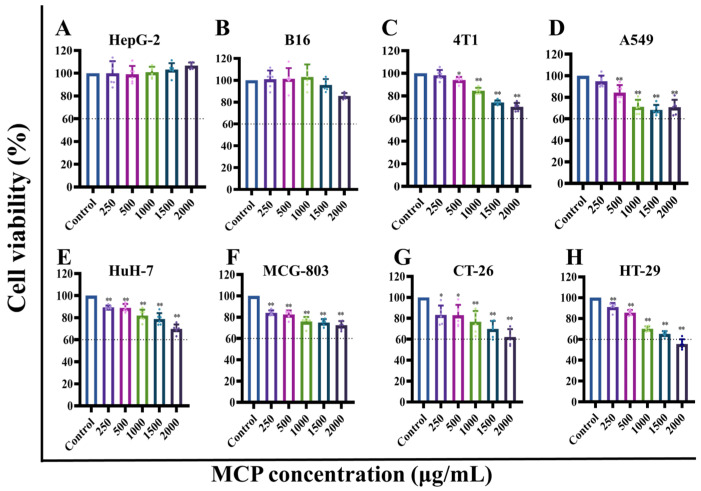
The impact of MCP on the survival of various cell types: (**A**) HepG-2 cells; (**B**) B16 cells; (**C**) 4T1 cells; (**D**) A549 cells; (**E**) HuH-7 cells; (**F**) MCG-803 cells; (**G**) CT-26 cells; (**H**) HT-29 cells. Values are mean ± SD (*n* = 6). ** *p* < 0.01, * *p* < 0.05 compared to the control group.

CCK-8 assays were employed to evaluate the inhibitory effect of MCP on MCG-803, HepG-2, HuH-7, A549, HT-29, CT-26, 4T1 and B16 cells, and the results are shown in Figure 1. The sample without polysaccharide served as the control group. The MCP did not exhibit significant inhibitory effects on HepG-2 and B16 cells (Figure 1A,B), while it showed significant inhibitory effects on 4T1, A549, HuH-7, MCG-803, CT-26, HT-29 cells. (Figure 1C–F,H). As the concentration of polysaccharides increased, the inhibitory effect of the MCP on cellular activity also exhibited a dose-dependent relationship. Among them, the most obvious inhibitory effects were noted in HT-29 and CT-26 cell lines, with the cell survival rate dropping to less than 60% in the high-dose group. The results suggest that MCP has a greater role in inhibiting the proliferation of colorectal cancer. Subsequently, anticancer activity studies were conducted on HT-29 cells.

#### 3.1.2. MCP-Attenuated Colony Formation and Migration Capacity in HT-29 Cells

**Figure 2:**
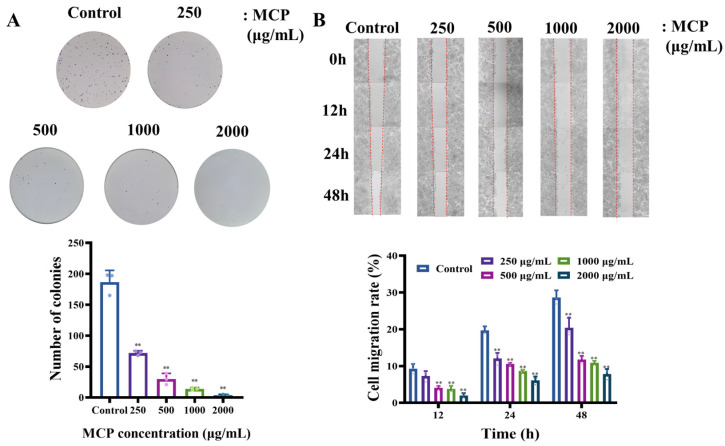
MCP reduces cell colony generation and migration of HT-29 cells. (**A**) Colony formation assay. (**B**) Wound healing. Experiments were conducted using three independent biological replicates, and representative images data are shown. ** *p* < 0.01 compared to the control group.

Colony formation assay is employed to evaluate cell survival by assessing the ability of a single cell to proliferate into a colony, which is a critical factor in determining the capacity for tumorigenesis exhibited by cancer cells in vitro [25]. The scratch assay, on the other hand, is used to assess the impact of a treatment on cell migration [26]. The findings of our study indicated a significant reduction in the colony numbers of cells treated with MCP in comparison to the control group. Compared with the control group, the number of cell colonies in the high-dose MCP group decreased by about 97% (Figure 2A).

Additionally, we examined the effects of MCP on the process of wound healing, finding that MCP treatment significantly impeded the healing of HT-29 cells. After the treatment with MCP, the mobility rate decreased from 19.4% to 5.81%, as shown in the wound healing assay (Figure 2B). The results collectively highlight the substantial impact of MCP on HT-29 tumorigenesis.

#### 3.1.3. MCP Induced Apoptosis in HT-29 Cells

**Figure 3:**
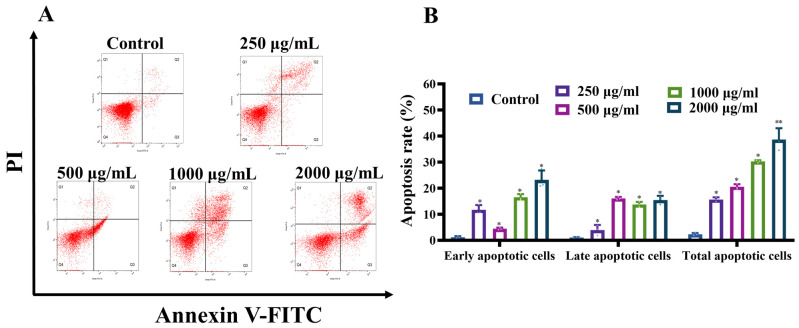
Effect of MCP on apoptosis of HT-29 cells. (**A**) Flow cytometry chart illustrating the apoptotic degree of HT-29 cells treated with MCP; (**B**) bar chart illustrating the percentage of apoptosis in HT-29 cells. Values are mean ± SD (*n* = 3). ** *p* < 0.01, * *p* < 0.05 compared to the control group.

To assess the extent of apoptosis in HT-29 cells induced by MCP, the cells were subjected to Annexin V-FITC/PI double staining. This method enabled a precise quantification of the proportion of apoptotic cells, which was subsequently analyzed using flow cytometry. Treatment with MCP for 48 h led to a gradual increase in the number of apoptotic cells compared to the basal apoptotic percentage in the untreated groups (Figure 3A). With the increase in MCP concentration, the apoptosis rate increased from 2.34% to 38.66% (Figure 3B). The findings suggested that MCP significantly promotes HT-29 cells apoptosis and suppresses cell proliferation in a dose-dependent manner.

#### 3.1.4. MCP-Induced Loss of Mitochondrial Membrane Potential (Δ*ψm*) in HT-29 Cells

We investigated whether MCP could cause the loss of Δ*ψm* in HT-29 cells by measuring mitochondrial membrane polarity using the JC-1 mitochondrial probe. The increase in the mitochondrial membrane is one of the main events in apoptosis; Δ*ψm* is an indicator of the first stages of cell apoptosis [27]. Furthermore, mitochondrial dysfunction typically initiates the apoptotic signaling pathway within the cell [28]. The noted decrease in red fluorescence, coupled with an increase in green fluorescence, indicates modifications in mitochondrial membrane permeability and a reduction in Δ*ψm* [29].

**Figure 4:**
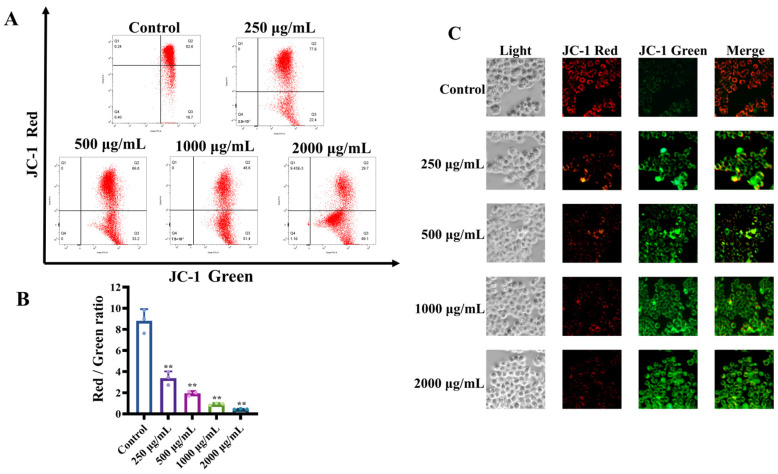
Effect of MCP on changes in mitochondrial membrane of HT−29 cells. (**A**) The mitochondrial membrane potential (Δ*ψm*) was tested; (**B**) bar charts of Δ*ψm* changes; (**C**) Δ*ψm* disruption in HT−29 cells imaged by inverted fluorescence microscope (40×). ** *p* < 0.01 compared to the control group.

As the concentrations of MCP increased, the percentage of red fluorescence in HT-29 tumor cells dropped dramatically from 82.3% to 31.03% (Figure 4A). Concurrently, the red/green ratio experienced a significant decline from 3.39 to 0.45, indicating that mitochondrial membrane depolarization was occurring in a concentration-dependent manner (Figure 4B). The control group exhibited red fluorescence within the mitochondria, while the green fluorescence displayed a dose-dependent increase following treatment with various concentrations of MCP (Figure 4C). This observation further supports the notion that the mitochondrial membranes experienced depolarization. Consequently, these findings imply that the mitochondria play a significant role in apoptosis induced by MCP.

#### 3.1.5. Effects of MCP on the Expression of Genes with the Apoptotic Pathway

**Figure 5:**
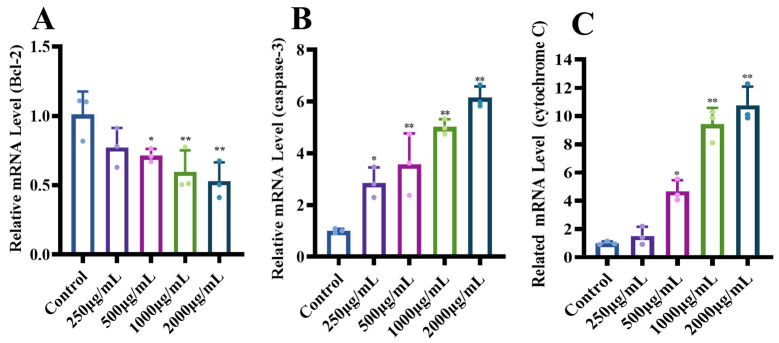
Effect of MCP on mitochondrial apoptosis pathway. (**A**) Expression of Bcl-2 gene; (**B**) expression of caspase-3 gene; (**C**) expression of cytochrome C gene. ** *p* < 0.01, * *p* < 0.05 compared to the control group.

In order to explore the possible mechanisms underlying MCP-induced apoptosis in HT-29 cells, we assessed the expression levels of apoptotic genes, including Bcl-2, caspase-3 and cytochrome C genes, involved in the mitochondria-dependent pathway using RT-qPCR. The MCP treatment could downregulate the expression of anti-apoptotic gene Bcl-2 compared to the control group with the increase in MCP concentration (Figure 5A). In contrast, the levels of cytochrome C and caspase-3 levels increased with increasing MCP concentration (Figure 5B,C). These results suggest that the MCP promoted apoptosis in HT-29 cells, by downregulating the anti-apoptotic gene Bcl-2, leading to the release of a large amount of cytochrome C, which in turn activated Caspase-3 [30], and triggered apoptosis of the cells through the mitochondrial pathway.

### 3.2. Immunomodulatory of MCP in RAW 264.7 Macrophages

#### 3.2.1. Impacts of MCP on RAW 264.7 Macrophages

**Figure 6:**
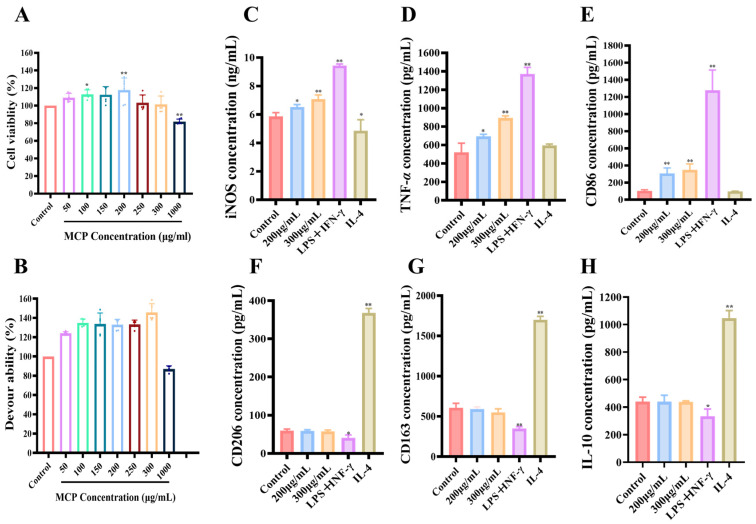
Effects of MCP on the activity, phagocytosis and polarization of original RAW 264.7 macrophages. (**A**) MCP on the viability of RAW 264.7 macrophages. (**B**) MCP on neutral red uptake by RAW 264.7 macrophages. Values are mean ± SD (*n* = 6). Effects of MCP treatment on the secretion of (**C**) iNOS, (**D**) TNF-*α*, (**E**) CD86, (**F**) CD206, (**G**) CD168 and (**H**) IL-10. Negative control, RAW 264.7 macrophages without MCP treatment; positive controls, RAW 264.7 macrophages treated with LPS + INF-*γ* and IL-4. Values are mean ± SD (*n* = 3). ** *p* < 0.01, * *p* < 0.05 compared to the control group.

The stimulation of the immune system relies heavily on the activation of macrophages. Research indicates that MCP demonstrates nontoxic properties toward RAW 264.7 macrophages when administered in concentrations between 50 and 1000 μg/mL. Notably, MCP markedly increased the viability of these macrophages at concentrations of 150–200 μg/mL (Figure 6A). On the flip side, a concentration of 1000 μg/mL of MCP inhibited the proliferation of RAW 264.7 macrophages. These results suggest that MCP effectively promoted macrophage proliferation within a certain concentration.

#### 3.2.2. Impacts of MCP on Phagocytosis Assay of RAW 264.7 Macrophages

The phagocytic function of macrophages is one of the most important non-specific immune responses in the body. Upon stimulation, macrophages are capable of phagocytizing some dyes such as neutral red in vitro [31].

RAW 264.7 macrophages were pretreated with MCP at different concentrations, and neutral red uptake was monitored to assess their pinocytic activity. After treatment with MCP for 24 h, RAW 264.7 macrophages showed significantly increased pinocytic activity as compared to the control group (Figure 6B). Up to 300 g/mL, this effect was dose-dependent. A further increase in pinocytic activity was not observed in macrophages treated with 1000 g/mL MCP. Based on both cell activity and phagocytosis activity, 200 and 300 mg/mL MCP concentrations were selected.

#### 3.2.3. Impact of MCP on Polarization of Original RAW 264.7 Macrophages

Previously, CD86 and iNOS have been found to be biomarkers for M1 macrophages, whereas CD206, CD163 and IL-10 have been found to be biomarkers for M2 macrophages [32]. To differentiate between M1 and M2 macrophages, we assessed these markers. Stimulation of RAW 264.7 macrophages with LPS + INF-*γ* led to a significant increase in the secretion of iNOS, TNF-*α* and the expression of CD86 compared to controls (Figure 6C–E). Notably, no significant changes were observed in IL-10 secretion or the expression of CD206 and CD168 in response to LPS + INF-*γ*, suggesting a shift towards the M1 phenotype (Figure 6F–H). Conversely, treatment with IL-4 resulted in a distinct pattern, indicating polarization towards the M2 phenotype.

The levels of TNF-α and iNOS secretion, along with the expression of CD86, were observed to increase in RAW 264.7 macrophages as the concentration of MCP was elevated from 200 to 300 μg/mL (Figure 6C–E). However, the secretion of interleukin-10 (IL-10) and the expression levels of CD206 and CD163 were not influenced by the presence of MCP, aligning with control levels (Figure 6F–H). These results suggested that MCP promoted the M1 polarization of macrophages, while M2 polarization was not influenced.

Furthermore, while levels of CD86 were similar in the 200 and 300 μg/mL MCP groups (Figure 6E), the secretion levels of iNOS and TNF-*α* were significantly different (Figure 6C,D). Specifically, the 300 μg/mL MCP treatment group presented a more pronounced effect. Notably, macrophages treated with 300 μg/mL MCP exhibited lower secretion levels of iNOS, TNF-*α* and CD86 when compared to those treated with LPS + INF-*γ*. These results suggested that MCP elicited a moderate M1 polarization response in the original macrophages without causing any inflammatory effects.

#### 3.2.4. Impacts of MCP on M2 RAW 264.7 Macrophages

**Figure 7:**
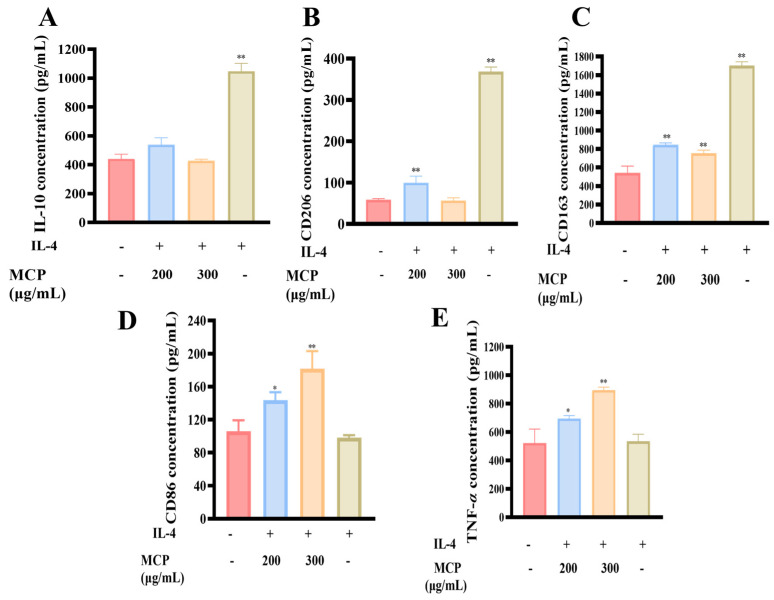
Effect of MCP on M2 macrophage RAW 264.7. Effects of MCP treatment on the secretion of (**A**) CD206, (**B**) CD168, (**C**) IL−10, (**D**) CD86 and (**E**) TNF−*α* in M2 RAW 264.7 macrophages treated with IL−4. Values are mean ± SD (*n* = 3). ** *p* < 0.01, * *p* < 0.05 compared to the control group.

Stimulation with IL-4 led to the polarization of M2 macrophages, as evidenced by the upregulation of CD206 and CD163 and an increase in IL-10 secretion. Interestingly, when MCP was co-administered with IL-4, a dose-dependent reduction in IL-10 secretion and a decrease in CD206 and CD163 surface markers were observed in the M2 macrophages, contrasting with the effects of IL-4 treatment alone (Figure 7A–C). Furthermore, the combined treatment of MCP and IL-4 resulted in an increase in CD86 expression, a distinct marker of M1 polarization, and enhanced TNF-*α* release, suggesting a shift towards a more pro-inflammatory phenotype (Figure 7D,E).

#### 3.2.5. Transwell Co-Culture System of RAW 264.7 and CT-26

**Figure 8:**
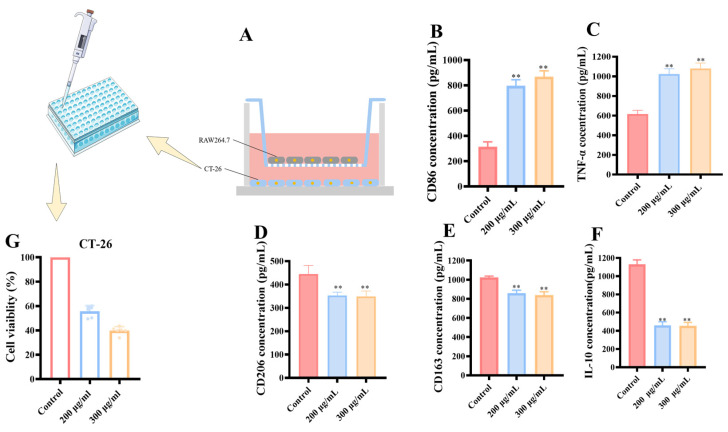
Effect of MCP in the co-culture system. (**A**) Schematic diagram of co-culture systems. Effects of MCP treatment on the secretion of (**B**) CD86, (**C**) TNF-α, (**D**) CD206, (**E**) CD168 and (**F**) IL-10 in RAW 264.7 and HT-29 Co-culture. Values are mean ± SD (*n* = 3). (**G**) Effects of MCP on the viability of CT-26 macrophages. ** *p* < 0.01 compared to the control group.

The above experimental data demonstrated that MCP effectively promoted M1 polarization in primary macrophages and induced a shift from M2 to M1 macrophages, resulting in a reduction in M2-polarized macrophages. To accurately simulate the tumor microenvironment (TME), a Transwell co-culture system was utilized to evaluate the effects of ERPP on the balance between M1 and M2 macrophages (Figure 8A). Since RAW 264.7 is a macrophage cell line derived from murine tissue, the isogenic intestinal cancer cell line CT-26 was employed for the Transwell assays. Treatment with MCP led to an increase in CD86 expression and TNF-α secretion, indicative of enhanced M1 polarization, while reducing CD206 and CD163 expression along with IL-10 secretion (Figure 8B–F), thus supporting a shift towards a more anti-tumor immune response. This modulation induced by MCP could potentially counteract the immunosuppressive milieu within tumors, positioning MCP as a promising candidate for cancer immunotherapy. In the co-culture system, the addition of MCP resulted in increased lethality of CT-26 cells compared to direct exposure, highlighting the enhanced anti-cancer effect of MCP in a more physiologically relevant setting (Figure 8G). The findings emphasize the ability of MCP to promote the transition of macrophage phenotypes, leading to an increased population of anti-tumor M1 macrophages while reducing M2 macrophages that aid tumor evasion. This modulation of macrophage polarization contributes to the anticancer effect.

### 3.3. Antioxidant Activities of MCP

#### 3.3.1. ABTS^•+^, DPPH• and Hydroxyl Free Radical Scavenging Analysis

**Figure 9:**
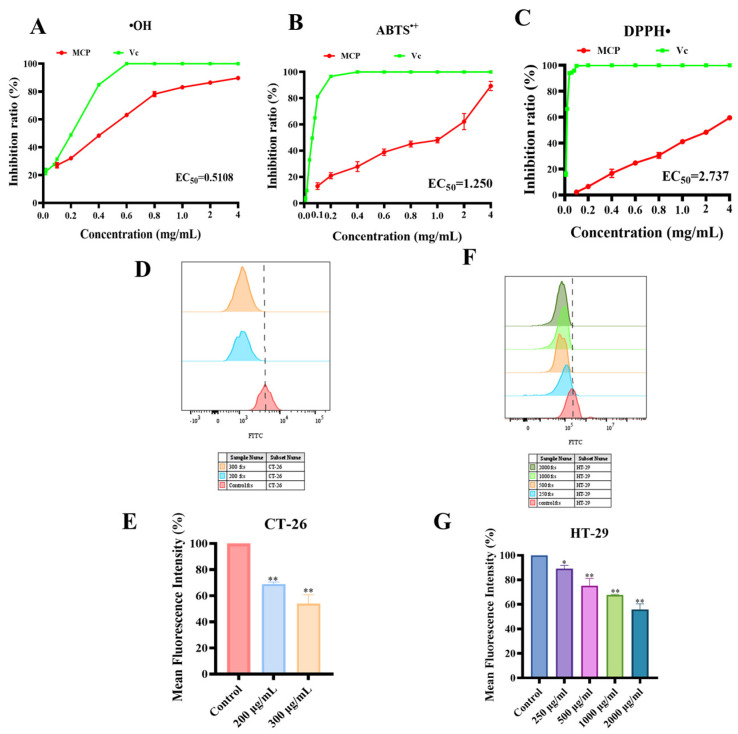
Antioxidant capacity of MCP. MCP exhibited antioxidant inhibitory effects on (**A**) hydroxyl radicals; (**B**) ABTS^•+^ and (**C**) DPPH•. Effect of MCP on the ROS of CT−26 (**D**) and RAW 264.7 in co-culture, and HT−29 alone (**F**), and its MFI value (**E**,**G**). Values are mean ± SD (*n* = 3). ** *p* < 0.01, * *p* < 0.05 compared to the control group.

The antioxidant activity of MCP was assessed through the use of ABTS^•+^, DPPH•, and hydroxyl free radical scavenging analyses, employing Vc as a positive control. MCP exhibited significant radical scavenging activity over a concentration range of 0.1 mg/mL to 4 mg/mL, showing a dose-dependent response. The ABTS ^+^ method measures the total antioxidant capacity [33], while DPPH• radical is a relatively stable free radical with a single electron [34]. Hydroxyl free radicals, on the other hand, are reactive oxygen species that can cause oxidative damage to cell tissue, causing disease [35]. The antioxidant capacity of MCP increased in a dose-dependent manner. Specifically, MCP exhibited superior ABTS^•+^ and hydroxyl radical scavenging abilities (EC_50_: 0.5108 and 1.25 mg/mL, respectively), with nearly 90% clearance rate at a concentration of 4 mg/mL (Figure 9A,B). In contrast, DPPH• scavenging activity was relatively weak, not exceeding 60% clearance in the measured concentration ranges (EC_50_ = 2.737 mg/mL) (Figure 9C).

#### 3.3.2. Effect of MCP on Intracellular ROS

ROS represent a group of short-lived highly reactive molecules formed intracellularly from molecular oxygen. Their effects on tumor cells and the immune system are concentration-dependent. Based on our above investigations, it is apparent that MCP exhibits notable efficacy in scavenging hydroxyl radicals. Within the physiological range, elevated ROS in immune cells can promote metabolism, synthesis and secretion of cytokines and other effectors [36]. To mimic the intricate milieu of the TME, we employed the Transwell chamber to assess the change in ROS levels following MCP treatment. As depicted in Figure 9, there was a noticeable and dose-dependent decline in the ROS mean fluorescence intensity (MFI) in CT-26 cells in the Transwell chamber treated with MCP (Figure 9D,E). The levels of reactive oxygen species (ROS) in HT-29 cancer cells were assessed, revealing a dose-dependent reduction as illustrated (Figure 9F,G). These findings are generally consistent with the results observed in CT-26 cells.

## 4. Discussion

Polysaccharides, known for their diverse pharmacological activities, stand out as versatile natural compounds with a wide range of therapeutic potentials [37]. Although previous studies have highlighted the inhibitory effects of polysaccharides on cancer cells and their significant immunomodulatory properties, particularly within the TME, much of the focus has been on elucidating these specific attributes. For instance, sea cucumber polysaccharides have been demonstrated to induce apoptosis in A549 cells and exhibit potent anti-tumor activity [38], In contrast, Astragalus polysaccharide, showed non-cytotoxicity towards 4T1 cells while activating macrophages to combat cancer cells effectively [39]. This study shifts the focus towards exploring the anticancer properties and immunomodulatory capabilities of MCP, a polysaccharide derived from *R. philippinarum* using fast extraction by microwave. The findings underscore the dual functionality of MCP in directly triggering apoptosis in HT-29 cells and modulating the polarization of macrophages towards the M1/M2 phenotype within the TME, thereby enhancing anticancer responses. In addition, MCPs also exhibit a certain antioxidant capacity, which further contributes to their anti-cancer activity. This suggests that MCPs may have a beneficial effect on the treatment of colorectal cancer.

Apoptosis serves as a crucial pathway for the regulation of tumor cell death in response to cytotoxic drugs [30]. The polysaccharide OP-1, derived from oysters, has been shown to inhibit the growth of HepG-2 cells in a time-dependent manner by inducing apoptosis [9]. Zhang RJ et al. demonstrated that abalone polysaccharide AVP induces apoptosis in MGC-803 cells in a dose-dependent manner, primarily through the downregulation of Bcl-2 and VEGF, and the upregulation of BAX and p53 expression [10]. Furthermore, our recent findings suggest that MCP enhances the rate of apoptosis as indicated by changes in mitochondrial membrane potential and Annexin V-FITC/PI staining.

The intrinsic apoptotic pathway, a classical cascade in apoptosis regulation primarily governed by the Bcl-2 family, is activated in response to external stimuli. Reduced expression of Bcl-2 disrupts mitochondrial integrity, leading to the excessive release of cytochrome C and subsequent formation of apoptotic bodies, which activates the caspase family and ultimately drives the apoptotic response [40]. Research on starfish polysaccharides has demonstrated their inhibitory effects on the metastasis of HT-29 human colorectal cancer cells [41]. This aligns with the findings of MCP research. The F2.1 polysaccharide, derived from *Donax variabilis* clam, has been found to upregulate apoptosis-related proteins, including Bcl-2, cytochrome C, cleaved caspase-3 and 9, while downregulating Bcl-2 protein levels, ultimately inducing cell apoptosis [42]. Our investigation into the molecular mechanisms underlying mitochondrial-mediated apoptosis revealed downregulation of the Bcl-2 gene alongside upregulation of cytochrome C and caspase-3 genes, consistent with previous findings in sea clams. These results suggest that MCP induces apoptosis in HT-29 cells by modulating the Bcl-2 gene, disrupting mitochondrial membrane potential, and promoting the formation of cytochrome C-induced apoptotic bodies, culminating in caspase activation and apoptotic progression.

The TME encompasses a specialized milieu surrounding tumor tissues, composed of various non-cancerous components including immune cells, fibroblasts, macrophages, capillaries, and the extracellular matrix [43]. Within this environment, tumor-associated macrophages (TAMs) emerge as a predominant immune cell population, predominantly adopting a tumor-promoting M2-like phenotype, which significantly influences tumor progression, metastasis and resistance to immunotherapy [44]. Notably, the transition from M1 activation during early tumor stages to an M2-like phenotype as tumor progresses promotes the immune escape of tumor cells [45]. Numerous investigations have explored the immunomodulatory effects of polysaccharides in the TME. Studies have revealed that polysaccharides such as MSGA from sea urchin eggs can activate signaling pathways including NF-kb and MAPKs (ERK1/2 and JNK), leading to the upregulation of inflammation-related enzymes and pro-inflammatory mediators, ultimately polarizing macrophages towards the M1 phenotype [11]. The polysaccharide SF-2, derived from *Asterias rollestoni*, regulates immunity by upregulating the levels of pro-inflammatory cytokines and related proteins, such as TNF-α and iNOS [46]. This finding was consistent with the results demonstrated by MCP. The present study suggests that MCP exhibits notable immunomodulatory activity on the TME. Treatment with MCP effectively reverses the M2 phenotype in the TME, promoting the polarization of macrophages towards the M1 type. Moreover, in a co-culture system, MCP-treated TAMs significantly enhance killing efficacy on tumor cells.

Numerous marine Mollusca polysaccharides exhibit notable antioxidant properties [47], with MCP also demonstrating particularly effective antioxidant capabilities in neutralizing hydroxyl radicals. Recent investigations have underscored the pivotal role of ROS within the tumor microenvironment. Tumor cells release substantial amounts of ROS into the TME, elevated ROS levels can trigger oncogenic pathways, thereby promoting tumor progression and metastasis [48]. Furthermore, heightened ROS levels can detrimentally impact TAMs within the TME, leading to immunosuppression and facilitating phenomena such as tumor immune evasion [49]. In this study, the assessment of ROS levels in co-cultured CT-26 cells revealed a significant reduction in ROS content within the TME following treatment with MCP. Previous studies have underscored the intimate association between ROS release and mitochondrial function. Consistent with these findings, our results suggest that MCP induces apoptosis in HT-29 cells, potentially through the intrinsic mitochondrial apoptotic pathway. Subsequent evaluation of ROS expression in MCP-treated HT-29 cells corroborated the findings from the Transwell chamber experiment. In summary, these findings support the antioxidant properties of MCP, effectively lowering the levels of ROS released by cancer cells into the TME, which may help inhibit immune evasion by tumor cells. Furthermore, since mitochondria are the primary organelles responsible for ROS production, the observed reduction in ROS levels in cancer cells may be associated with MCP-induced mitochondrial apoptosis.

## 5. Conclusions

The polysaccharide MCP, extracted by microwave, inhibited the growth of HT-29 cells without causing significant toxicity in RAW 264.7 cells. MCP is capable of inducing apoptosis in HT-29 cells, potentially through mechanisms that reduce intrinsic mitochondrial membrane potential, facilitation of cytochrome C release, activation of caspase-3 and suppression of Bcl-2 gene expression. Moreover, MCP demonstrates the capability to reverse the polarization of M2-type TAMs to M1-type within the TME, as well as promoting the differentiation of original macrophages into M1-type cells, thereby enhancing their anti-cancer effects. MCP also reduces ROS levels in tumor cells. These results emphasize the importance of conducting further research on MCP as a potential safe, functional food and as natural anti-tumor and immunomodulatory agents.

## Figures and Tables

**Table 1:** Primers sequence of apoptotic genes.

Gene	Primer Sequence	
*Bcl-2*	F	ATCGCCCTGTGGATGACTGAGT
	R	GCCAGGAGAAATCAAACAGAGGC
*Caspase-3*	F	AGAGGGGATCGTTGTAGAAGTC
	R	ACAGTCCAGTTCTGTACCACG
*Cytochrome C*	F	CTTTGGGCGGAAGACAGGTC
	R	TTATTGGCGGCTGTGTAAGAG
*β* *-actin*	F	GGGACCTGACTGACTACCTC
	R	TCATACTCCTGCTTGCTGAT

## Data Availability

The original contributions presented in the study are included in the article and Supplementary Materials, further inquiries can be directed to the corresponding author.

## Supplementary Materials

The following supporting information can be downloaded at: https://www.mdpi.com/article/10.3390/foods13223552/s1, Figure S1: The monosaccharide composition of MCP. (A): Standard sugar liquid chromatogram. (B): MCP liquid chromatogram. Table S1: Percentage of each component (%) in MCP.
