# Supplementary material for: Evaluation of Anticancer and Immunomodulatory Effects of Microwave-Extracted Polysaccharide from Ruditapes philippinarum
Source: Foods. 2024 Nov 7;13(22):3552. doi: 10.3390/foods13223552 (PMC11593841; doi:10.3390/foods13223552)
Supplement: Supplementary file 1 [file foods-13-03552-s001.zip › foods-3258013-supplementary.pdf]

Supplementary material

Evaluation of Anticancer and Immunomodulatory Effects of Microwave-Extracted Polysaccharide from *Ruditapes philippinarum*

Mengyue Liu [1], Fei Li [1], Shuang Feng [2 3], Jiamin Guo [2 3], Jia Yu [1,*], Shengcan Zou [2 3], Xiang Gao [1] and Yuxi Wei [1,]

1 College of Life Sciences, Qingdao University, Qingdao 266071, China; lmy02112021@163.com (M.L.), pdlifei@163.com (F.L); xx526916212@126.com (X.G)
2 Qingdao Yihai Industry Holdings Co., Ltd., Qingdao 266105, China; m18565444972@163.com (S.F); gjm-study@163.com (J.G); zoushengcan@chenland.cn (S.Z.)
3 Qingdao Chenlan Pharmaceutical Co., Ltd., Qingdao, 266105, China; m18565444972@163.com (S.F); gjm-study@163.com (J.G); zoushengcan@chenland.cn (S.Z.)
* Correspondence: yujiaqdu@163.com (J.Y.).

Monosaccharide composition analysis

Monosaccharide standards (rhamnose, arabinose, galactose, glucose, xylose, mannose, glucuronic acid, galacturonic acid, glucosamine hydrochloride, and amino galactose hydrochloride monosaccharides) were accurately weighed to 5.0 mg each. After dissolution, the volume was adjusted to 10mL using a volumetric flask, and the solutions were diluted to 10, 20, 40, 60, 80, 100, 200, 500 μg/mL (fucose was diluted to 20, 40, 60, 80, 100, 200, 500, 1000 μg/mL). Standard curves were established by preparing standard samples of different concentrations. The samples were then treated with TFA, heated, and dried under nitrogen. Subsequently, sodium hydroxide solution and PMP methanol solution were added. After vortex mixing, the reaction was carried out in a water bath at 70°C for 1 h. The mixture was extracted with chloroform, which was discarded, and the remaining aqueous phase was adjusted to a final volume of 1 mL with water. To detect the monosaccharide composition of MCPs, a Thermo U 3000 liquid chromatography system (Thermo, USA) equipped with ZORBAX Eclipse XDB-C18 was used. The system operated with a flow rate of 0.8 mL/min, a column temperature of 30°C, a detection wavelength of 250 nm, an injection volume of 10 μL.

The MCP, as determined by the PMP-HPLC method, was primarily composed of glucose, arabinose, rhamnose, glucuronic acid, galacturonic acid, and mannose (Figure S1). The relative proportions of these components were detailed in table S1. The results of the aforementioned experiments indicated that glucose was the predominant constituent of MCP.

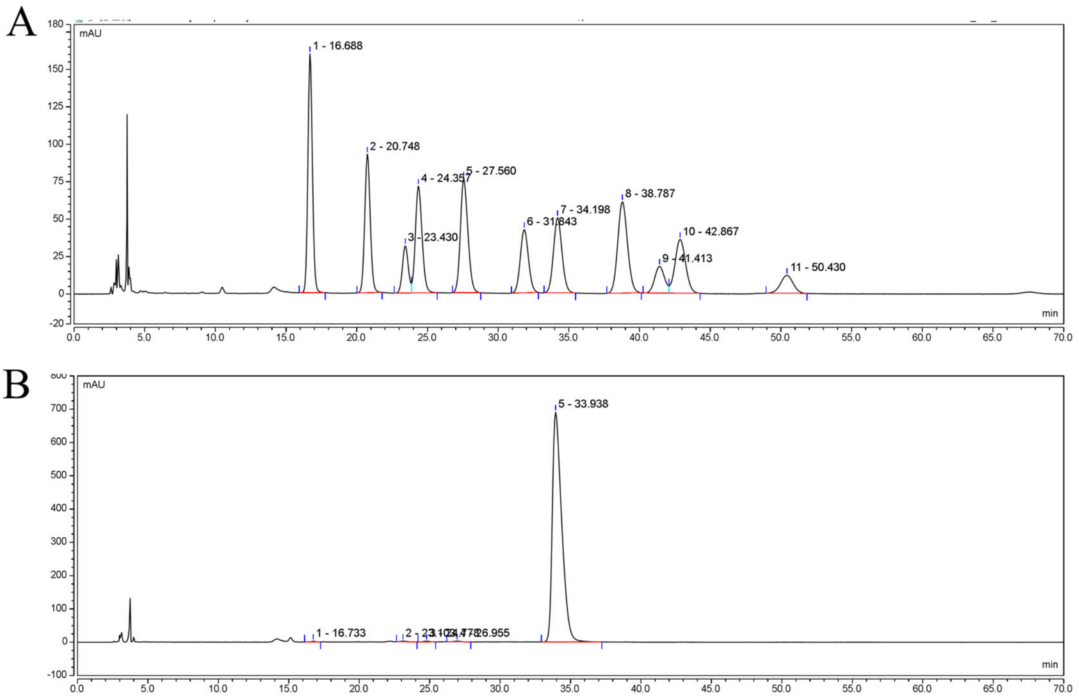

Figure S1: The monosaccharide composition of MCP. (A): Standard sugar liquid chromatogram. (B): MCP liquid chromatogram.

Table S1: Percentage of each component (%) in MCP

Percentage of each component (%)										
**Man**	**GlcN**	**Rha**	**GlcA**	**GalA**	**GalN**	**Glc**	**Gal**	**Xyl**	**Ara**	**Fuc**
0.099	0.00	0.268	0.258	0.167	0.00	92.345	0.000	0.000	6.863	0.000
